# Chemical Electron Microscopy (CEM) for Heterogeneous Catalysis at Nano: Recent Progress and Challenges

**DOI:** 10.34133/research.0043

**Published:** 2023-02-24

**Authors:** Yinghui Pu, Bowen He, Yiming Niu, Xi Liu, Bingsen Zhang

**Affiliations:** ^1^ Shenyang National Laboratory for Materials Science, Institute of Metal Research, Chinese Academy of Sciences, 72 Wenhua Road, Shenyang 110016, China.; ^2^ School of Materials Science and Engineering, University of Science and Technology of China, 72 Wenhua Road, Shenyang 110016, China.; ^3^ School of Chemistry and Chemical Engineering, In-situ Center for Physical Sciences, Shanghai Jiao Tong University, Shanghai 200240, China.

## Abstract

Chemical electron microscopy (CEM), a toolbox that comprises imaging and spectroscopy techniques, provides dynamic morphological, structural, chemical, and electronic information about an object in chemical environment under conditions of observable performance. CEM has experienced a revolutionary improvement in the past years and is becoming an effective characterization method for revealing the mechanism of chemical reactions, such as catalysis. Here, we mainly address the concept of CEM for heterogeneous catalysis in the gas phase and what CEM could uniquely contribute to catalysis, and illustrate what we can know better with CEM and the challenges and future development of CEM.

## Introduction

**Fig. 1.**
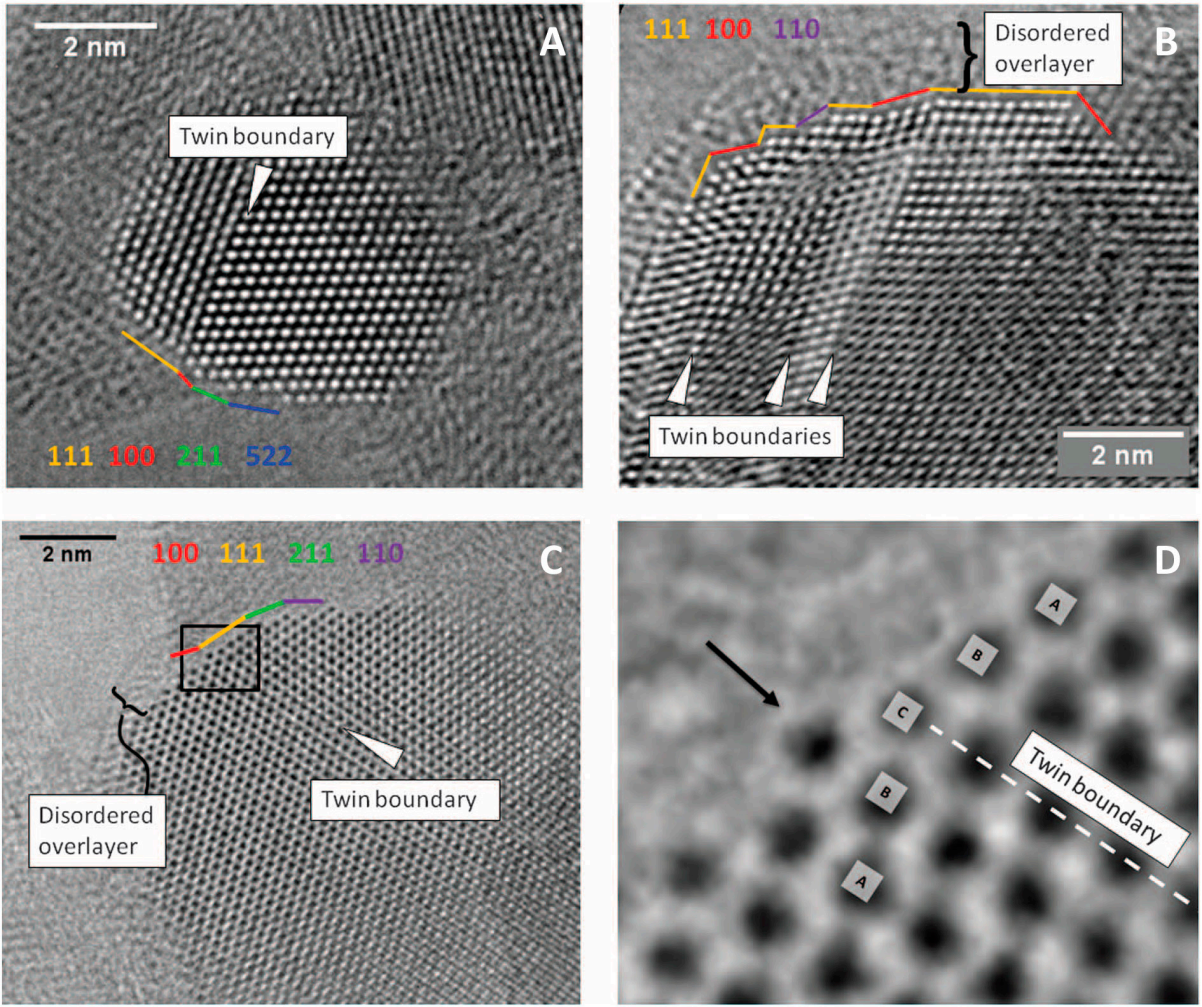
High-resolution TEM images of Cu NPs in the most active Cu/ZnO/Al_2_O_3_ catalyst (A to D), showing the bulk defects and surface steps. Reprinted with permission from [8].

**Fig. 2.**
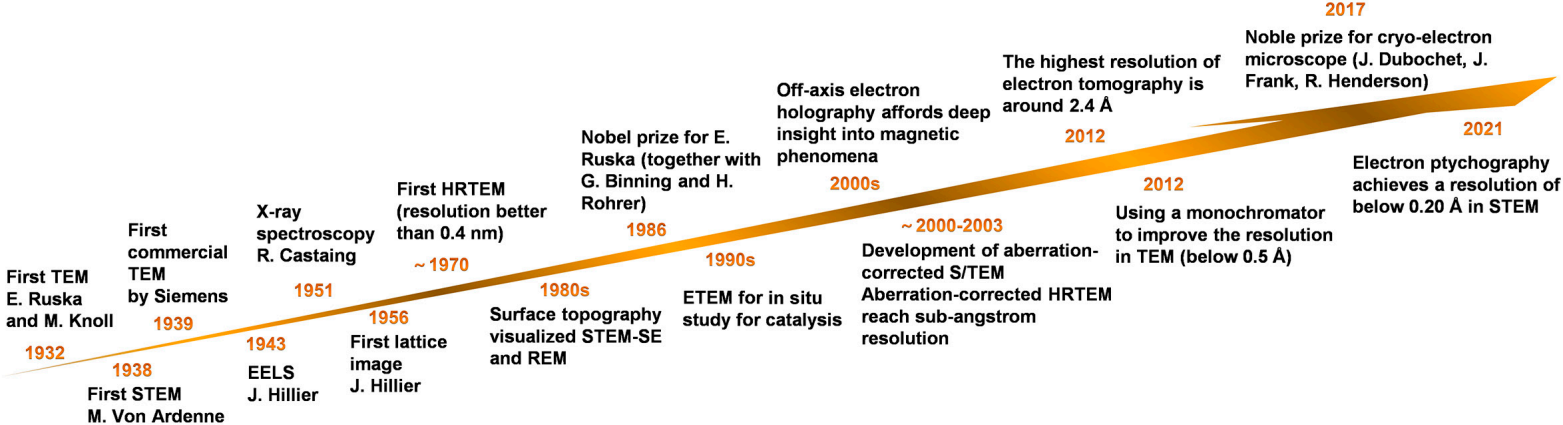
Some selected major events about the development of electron microscopy from 1930s to 2020s.

One aim of studying chemical transformations at the interface of nanomaterials by advanced characterization methods is to identify the working microstructures at molecular and mesoscopic dimensions to explore relevant structure–function relations. Transmission electron microscopy (TEM) is an effective characterization tool that enables us to directly observe and reveal the microstructures of materials [1]. In its earlier time, TEM was mostly used for the study of the defect structure of thin specimens [2]. This is possible since diffracted electrons can be used to form images in a TEM by various contrast mechanisms. The Howie–Whelan equation describing the transmissions of direct and diffracted electron beams laid the foundations for understanding diffraction contrast in TEM [3,4]. Linear defects (dislocations) and planar defects (translation boundary, grain boundary, phase boundary) can be analyzed in fine detail [5]. One example is the identification of stacking faults in the face-centered cubic (FCC) material by strong-beam images recorded by bright- and dark-field images using strongly excited direct or diffracted beam, respectively [6]. An example is Cu nanoparticles (NPs) used for the synthesis of methanol from CO_2_ and hydrogen exhibiting stacking fault and twin boundary imaged by TEM (Fig. 1) and quantified by neutron scattering [7,8]. These investigations revealed [9] the relevance of these defects for the catalytic function. Weak-beam dark-field microscopy [10] is a dedicated but powerful technique to image dislocations [11], particularly dissociated dislocations in materials [12]. While the diagnostic function of TEM in materials science to identify structure fault is indispensable, the well-established convergent beam electron diffraction (CBED), historically the oldest TEM diffraction technique, was developed by Kossel and Moellenstedt in 1938 [13], long before the well-known selected-area diffraction by Le Poole in 1947 [14]. It can be used to determine the structure by providing crystallographic information on unit cell, lattice parameters, crystal system, and 3-dimensional (3D) crystal symmetry (point group and space group). CBED is also a powerful tool to study strain in samples and to determine the specimen thickness [15]. Apart from these breakthroughs, many additional milestones in the history of TEM were essential to arrive at the present toolbox of sophisticated methods [1] in the family of electron microscopies indicated in Fig. 2. The Nobel Prize for Ernst Ruska in 1986 recognized this, although multiple game-changing inventions, namely, the correction methods for aberrations of electron lenses, contributed later to the toolbox.

The modern developments that revolutionized materials characterization and thus materials science that are relevant for the understanding of catalytic function are as follows.

(1) The availability of techniques to detect x-ray photons emitted from electrons transmitted through the specimen, the well-established energy-dispersive x-ray spectroscopy (EDX) [16], or the methods to analyze the inelastic scattering in the form of electron energy loss spectroscopy (EELS) [17]: The changes from serial to parallel recording of EELS and the implementation of 4 silicon drift detectors (SDDs) symmetrically around the objective lens pole pieces [18] are milestones that allow rapid and efficient acquisition of spectra before electron irradiation damage. This enables the reliable compositional analysis of a specimen and the determination of chemical and electronic state of its constituting elements [19]. Today, all these information can be obtained from individual atom columns or extremely small volumes of material, e.g., catalytic nanocomposites or even single atoms [20–23]. This is the well-established analytical electron microscopy (AEM) [24]. AEM is not a simple supplement of structural information obtained by diffraction and image technique. AEM can rather be used to measure charge transfer between 2 objects and determine crystal field splitting and dielectric and plasmonic properties of investigated materials. In addition, AEM has been developed as a platform for many other experiments that are otherwise only possible with photons. We mention here energy-loss magnetic chiral dichroism [25,26] and electron Compton scattering of solids [27,28].

(2) The implementation of aberration corrections, both on TEM and scanning TEM (STEM), allows the determination of positions of atoms in a solid with a precision of a few picometers [1]. The highlights are the image of light atoms such as H or Li column in a crystal or single atoms/clusters in/on a solid [29,30]. The aberration-corrected high-resolution TEM (HRTEM) and high-angle annular dark-field STEM (HAADF-STEM) have revolutionized the materials characterization in the past years, providing valuable information that otherwise cannot be achieved for many research disciplines extending from semiconductors to solar cells to catalysis [1,31–34]. In the present context, it is most relevant that aberration correction allows imaging the surface of a nanostructured solid [35] with atomic resolution without having to account for the disturbing contrast of the termination from a periodic array of atoms (Fig. 1) [8]. We can now detect surface terminations on complex objects [36–38] without having to retreat to planar model systems amenable to low-energy electron diffraction.

(3) Parallel to these developments is the implementation of electron holography, originally proposed by Dennis Gabor in 1948 [39] to correct aberration by wave interference and Nobel-prized in 1971 [40], electron tomography, implemented at first by Aaron Klug and Nobel-prized in 1982 for resolving the structure of nucleic acid–protein complexes, on a modern TEM [41], and electron ptychography, postulated by Hoppe in the 1960s and very recently established as an efficient method for imaging complex structure with a record-breaking resolution of just 0.2 Å [42–44]. One reason for the later implementation of the 2 techniques on TEM is the automatization of specimen holders (for tomography) or detectors (for ptychography) [41] and the computational capacities of personal computers.

(4) Along with the instrumental development, aberration-corrected scanning electron microscopy integrated with STEM has been realized, able to spontaneously obtain secondary electron (STEM-SE) images as well as annual dark-field images with sub-angstrom resolutions [45]. The technical advance allows precise determination of surface atomic structure of nanomaterials even under the realistic chemical environment [46].

One common point in applying TEM, AEM, HRTEM, and HAADF-STEM as well as electron holography and tomography is that they are used to study the static property/structure of a specimen. Although all the achievements mentioned above open new areas in materials science, for instance, the emergence of nanomaterials, in many cases the obtained information is only indicative, but not determinative. One example is functional materials: The property of a functional material depends on the function that is acting in a given chemical/biological/physical environment or condition. TEM operates in a high vacuum, which is not the usual condition that functional materials work.

**Fig. 3.**
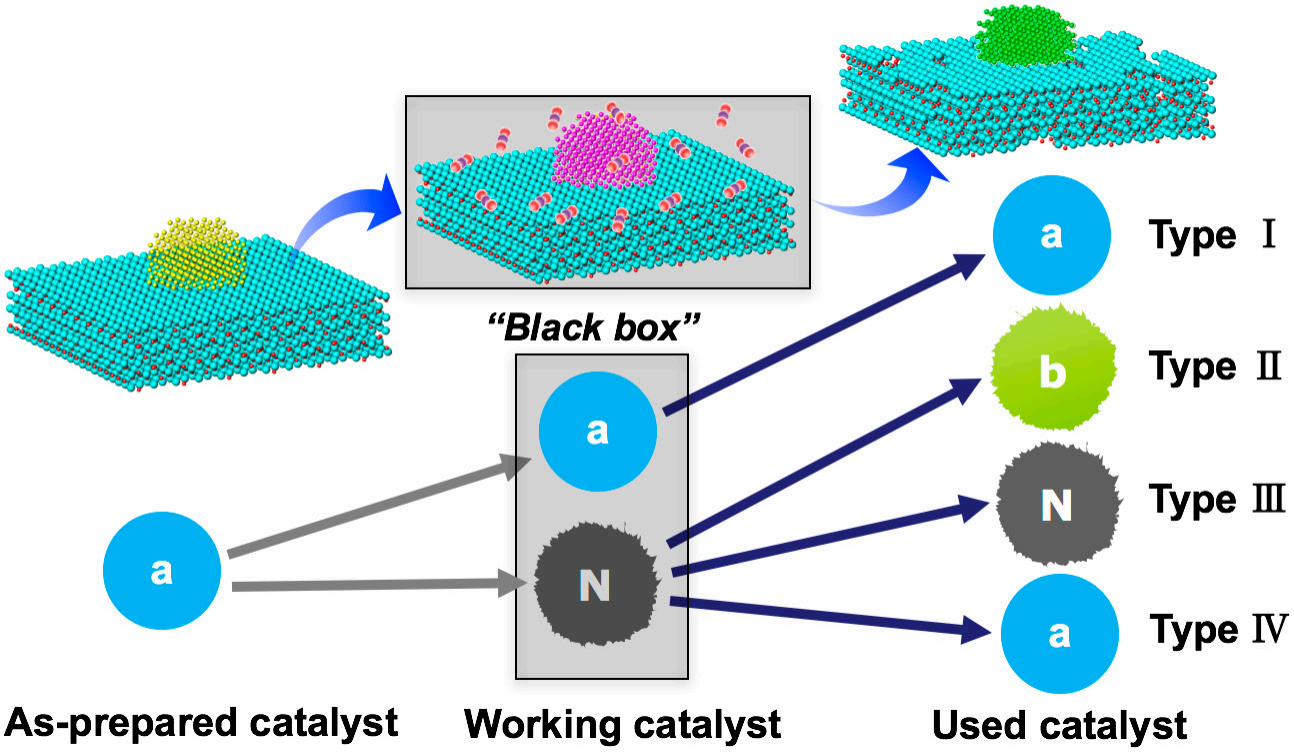
Options of structural evolution of a catalyst undergoing a chemical reaction.

Catalytic materials are among the mostly studied functional materials. It is the rule in most of the laboratories operating electron microscopes that studies on catalysts are carried out in high vacuum and at room temperature. As it is illustrated in Fig. 3, there are 4 types of catalyst depending how the catalyst behaves under and after reaction conditions: Type I is the catalyst that its structure does not suffer any structural changes during or after reactions. Type II catalyst has different states under working condition and after reaction. Type III catalyst is the one that the active phase of the catalyst is “frozen” or “quenched” after reaction. Type IV catalyst can have a dynamic state during the reaction but returned to the as-prepared state when applied chemical potential is removed. It is obvious that the best case is the type I catalyst; a study of fresh or used catalyst alone would be enough to establish structure–performance relationships exactly. Also, the investigation of type III is easier since a postmortem study of used catalyst would thus provide sufficient information about the structure of the catalyst under reaction conditions. However, these 2 types cannot be foreseen or predicted without support from other in situ experiments.

The critical yet most general cases are types II and IV, with the type IV being the worse, since a comparison of fresh and used catalyst could lead to the wrong conclusion that the catalyst might not suffer any structural dynamics during the reaction. This illustration reveals how relevant it is to establish a structure–function relation in a pre- or postmortem study. It is thus generally inadequate to derive structure–performance correlations from a simple comparison of catalyst structures before and after reaction. The exact state of the catalyst during a reaction can be distinct from those of fresh and used samples. The state during reaction (the “black box” in Fig. 3) is unknown but is the key and the reason for catalyst characterization with the aim to understand the function based on structural observations.

In fact, a working catalyst is a complex solid and its structural complexity is associated with the ability to undergo in every catalytic cycle regeneration of the active sites, as these sites are high energy elements in the solid matrix processes that reduce the local high energy accompanying the generation of active sites. These processes are phase transitions, segregations, reconstructions, leaching, deposition, or mobility on supports. Heterogeneous catalysis reactions occur at the interface of gas/solid or liquid/solid [20,47–52]. Moreover, we have to keep in mind that catalysts perform their function under transport of mass and energy. Differing from many other functional materials, catalysts are thus continuously confronted with environmental conditions (chemical potential) under which making and breaking of chemical bonds occur including between atoms that belong to the catalyst structure. In other words, a study is missing out on the most relevant part of being the reactive catalyst structure. It is not the “material” but the catalyst in reaction that should be studied if one is interested in its function under performance conditions. When this should be done at the atomic level, we need the novel toolbox of chemical electron microscopy (CEM).

## Chemical Electron Microscopy

The term CEM comprises all techniques of imaging and spectroscopy mentioned above, allowing to study an object (the reason we use here the word “object” instead of “catalyst’ will be explained later) in a given chemical environment under conditions of observable performance. In other words, CEM regards an object and the reactants/products under heat/mass transport as a system. Its inherent time resolution allows studying the dynamics of the system instead of only imaging the static structure of the object (or catalyst). The time resolution is very slow in units of elementary reactions but well sufficient under the limitations of material and heat transport through the specimen under observation. The focus here is still on the structure of the object and its response to the chemical environment. The kinetics of observable transformations can be determined locally and further compared to the kinetics of the transformation of the reactants available from conventional experiments. In this way, we envisage that complementary kinetic descriptions of catalytic reactions contain not only the terms for the transformation of the reactants but also those for describing chemical dynamics, giving rise to a steady state of active sites and those describing the chemical reactivity of deactivation following the pathways to minimize the excess energy of the catalyst. Both these phenomena are accessible in CEM if sufficient observations are available and if the genuine dangers of pre-occupation in selecting “typical regions” of interest are carefully observed.

CEM differs from in situ TEM since the term “in situ TEM” refers to its ability of capturing dynamic changes of objects under certain environments. Typical examples are heating experiments to study the thermal behavior of a specimen at elevated temperature or deformation by applying a force (bending, stretching, distorting) to study mechanical properties of the samples. Electron microscopy operates at high vacuum required by the electron gun but also due to the very small mean free path of electrons in any gas under ambient pressure.

**Fig. 4.**
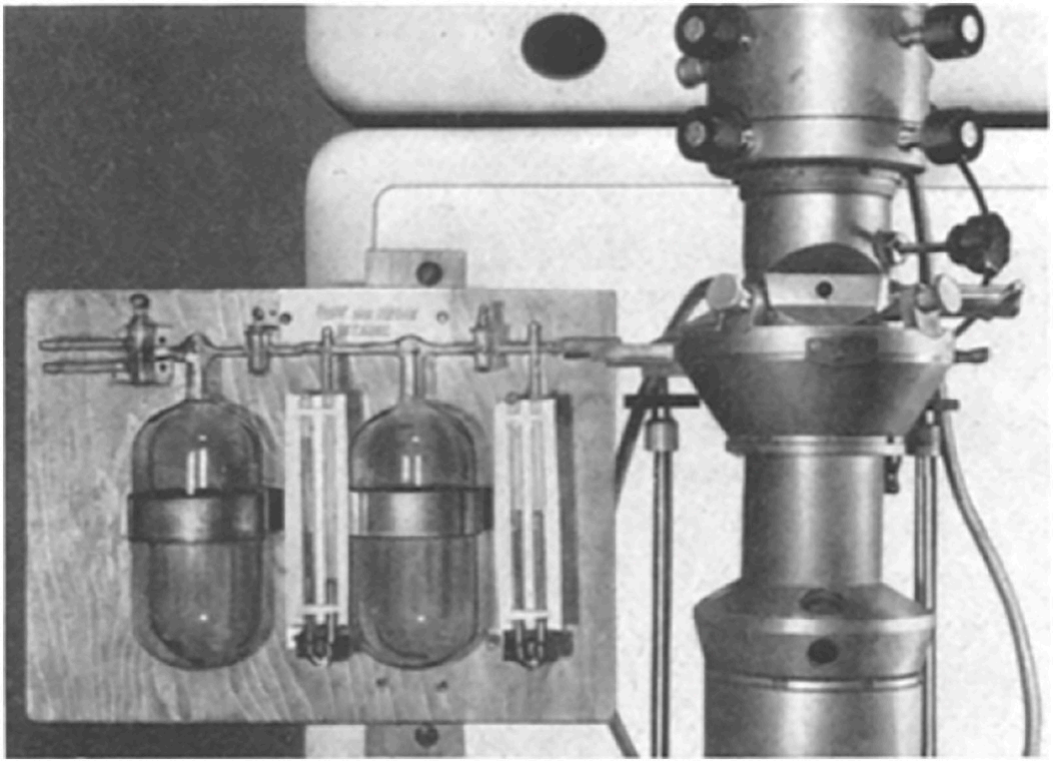
Photo of electron microscope for gas supply developed in 1942. Reprinted with permission from [53].

Understanding the response/behaviors of a specimen to/in its chemical environment with a high local resolution is the dream of materials scientists and chemists. Ernst Ruska, who co-invented the electron microscope in 1930s and received the Nobel prize in 1986, published in 1942 probably the first paper reporting imaging of silver colloidal particles at various stages of pressure from 10^−4^ to 40 torr and the transformation of colloidal silver to silver chloride (Fig. 4) [53]. In 1968, H. Hashimoto developed a high-temperature gas reaction specimen chamber for TEM [54]. It follows the pioneering work of Flower and Baker in the 1970s [55]. With the efforts of many groups, especially the works of Gai and Boyes, CEM, also called ETEM (environmental TEM) or ESTEM (environmental STEM), is becoming an established research tool in many laboratories [56,57]. In the following, we would like to illustrate the concept, development, and perspective of CEM. Solid catalysts are selected as the representative nanomaterial studied by CEM.

### Differential pumping aperture-type environmental cell

**Fig. 5.**
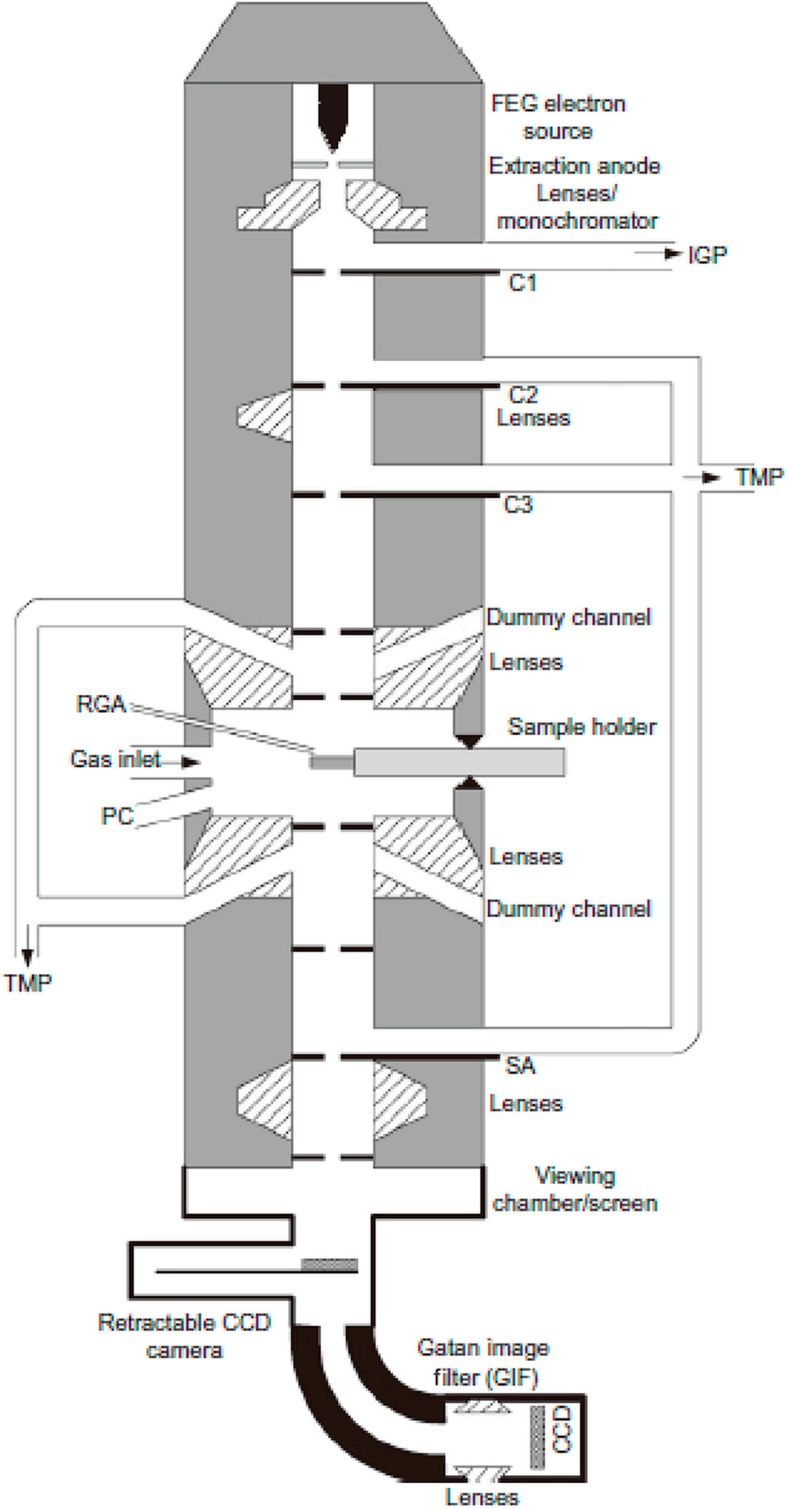
Schematic diagram shows a cross-section of a differential pumping aperture-type ETEM. FEG, field emission gun; IGP, ion getter pump; TMP, turbomolecular pump; RGA, residual gas analyzer; PC, plasma cleaner; SA, selected-area aperture; CCD, charge-coupled device. Reprinted with permission from [58].

This is to create a defined chemical potential around the specimen without disturbing the vacuum requirement in other parts of the TEM. To use this so-called differential pumping aperture-type environmental cell (ECELL), the TEM column has to be modified to install numerous differential pumping apertures and/or several sets of differential pumping vacuum systems between the gun and the viewing chamber (Fig. 5) [58]. The differential pumping aperture with a diameter of dozens of micrometers is used to create a poor vacuum area and high vacuum area by limiting diffusion of gases, but allows direct pass-through electron. Moreover, the pumping stage placed in the upper and lower parts of the objective lens pole pieces is close to the specimen holder and takes out most of gases diffusing out of the apertures. The second one is added between the condenser aperture and selected-area aperture for protecting the illumination system of the microscope. This is critical in field emission sources, which are preferred for CEM as their very narrow electron beam is least broadened by collisions with the gas surrounding the specimen. To ensure uncompromised operation of the gun, a third differential pumping unit is installed just below the illumination system. At present, the maximum pressure during observation has reached 5,000 Pa and the temperature may be up to 1,000 °C [59]. The resolution is reported to be 0.24 Å in Cs-corrected CEM (N_2_, 4,000 Pa; room temperature) operated at 300 kV [60].

### Window-type ECELL

**Fig. 6.**
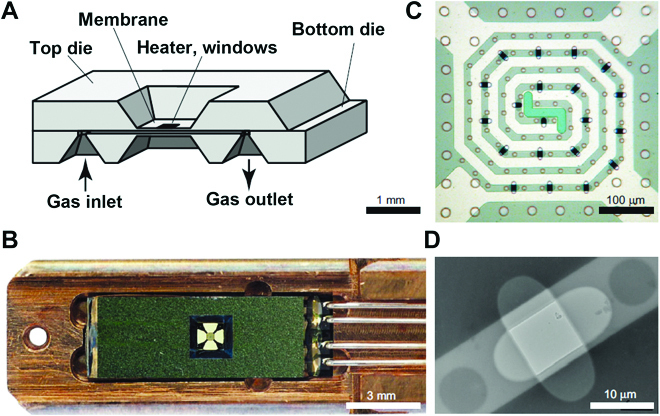
Illustration of a microelectromechanical system (MEMS) technology-based window-type ECELL. Schematic diagram of a nanoreactor (A), the TEM holder with the nanoreactor (B), the nanoreactor membrane (C), and a TEM image of a pair of superimposed 10-nm-thick windows. Reprinted with permission from [61].

An alternative to differential pumping aperture-type ECELL is the window-type ECELL (Fig. 6) [61,62], which is installed in a sample holder. The samples are placed within 2 thin inorganic windows (e.g., Si_3_N_4_, now also possible with graphene) that enable electron transmission. This window-type ECELL allows gases or liquids to surround the specimen in static and/or flow modes [61–65]. The very thin volume transmitted by the electron beam retains resolution. The windows substantially influence the contrast. Heating is possible through a resistive coil incorporated in the specimen support. The typical thickness of the gas layer separated from vacuum by 2 parallel 10-nm-thick Si_3_N_4_ windows is about 1 μm. The controlled gas environment in it can be realized at a pressure up to 4.5 bar and at a temperature up to 400 °C [62]. The resolution when using windowed ECELL is influenced by the thickness of the gas layer (i.e., the density of gases) and by the windows (structure, thickness) because the 2 membranes and the gas molecules scatter electrons and decrease the signal-to-noise ratio.

## CEM Case Studies

Table 1 summarizes some selected gas–environment studies in a CEM. Although the CEM technique is not broadly applied yet, interesting studies are reported in the literature highlighting the enormous added value for our understanding of catalyst function when investigated by CEM [66–72].

**Table 1.** Some typical environmental studies on gas atmosphere in a CEM.

Title	Reaction (or chemical process)	Experimental conditions	Classes of ETEM	Year [reference]
Beitrag zur übermikroskopischen Abbildung bei höheren Drucken	Interaction of colloidal silver with hydrogen and chlor	Air, H_2_, Cl_2_; ~20 °C; 200 torr		1942 [53]
High-temperature gas reaction specimen chamber for an electron microscope	The redox of some compounds (Mo_9_O_26_); growth process of Cu crystal	H_2_ and air; 1,000 °C; 300 torr	Gas reaction cell (DP)	1968 [54]
In situ electron microscopy studies of catalyst particle behavior	Carbon formation; carbon gasification; sintering of supported metal NPs (Co, Ni, Cr, Mo, etc.)	C_3_H_3_ and O_2_; 25–1,300 °C; up to 400 torr	CAEM (gas reaction cell) (DP)	1979 [55]
An atomic oxygen environmental cell for a transmission electron microscope	The interaction of atomic oxygen with graphite, filamentous carbon, and various polymers	H_2_, O_2_, and N_2_O; 1,000 °C, 2 torr H_2_ or 15 torr O_2_	ECELL (DP)	1990 [148]
Environmental high-resolution electron microscopy and applications to chemical science	Studying SMSI of Pt/Ti_2_O; transformation of the precursor VHPO to VPO ^a^	N_2_ and H_2_; >1,000 °C; 0–50 mbar	ECELL (DP)	1997 [56]
Atom-resolved imaging of dynamic shape changes in supported copper nanocrystals	The dynamic shape changes of supported Cu NPs in different atmosphere	H_2_, H_2_/H_2_O, and H_2_/CO; 220 °C; 1.5–50 mbar	ECELL (DP)	2002 [80]
Atomic-scale imaging of carbon nanofiber growth	Carbon nanofiber formation from methane decomposition over supported Ni NPs	H_2_ and H_2_/CH_4_; up to 540 °C; up to 35 mbar	ECELL (DP)	2004 [90]
Environmental electron microscopy (ETEM) for catalysts with a closed E-cell with carbon windows	Au and Pd supported on TiO_2_ and amorphous carbon (annealing in H_2_)	H_2_ and O_2_; up to 350 °C; up to 10 mbar	Windowed ECELL	2006 [149]
Direct observations of oxygen-induced platinum nanoparticle ripening studied by in situ TEM	Pt/Al_2_O_3_ catalyst ripening	Air; up to 900 °C; up to 15 mbar	ECELL (DP)	2010 [150]
Visualizing gas molecules interacting with supported nanoparticulate catalysts at reaction conditions	Au/CeO_2_ catalyzed CO oxidation	CO, O_2_, and N_2_; ~20 °C; 100 Pa	ECELL (DP)	2012 [151]
Visualization of oscillatory behavior of Pt nanoparticles catalyzing CO oxidation	Pt NPs catalyzed CO oxidation	CO, O_2_, and He; 520 °C; 1 bar	MEMS-based ECELL	2014 [124]
Environmental transmission electron microscopy (ETEM) studies of single iron nanoparticle carburization in synthesis gas	Fe NP carburization in synthesis gas	CO and H_2_; 280 °C; 6.6 mbar	MEMS-based ECELL (DP)	2017 [152]
Structural changes in noble metal nanoparticles during CO oxidation and their impact on catalyst activity	CO induced structure change of noble metal NPs (Pd, Pt, and Rh)	CO and O_2_; 200–600 °C; 760 torr	MEMS-based ECELL	2020 [126]
In situ manipulation of the active Au–TiO_2_ interface with atomic precision during CO oxidation	Interaction between Au and TiO_2_ modulated by CO oxidation	CO, O_2_; 500 °C; 1 bar	MEMS-based ECELL (DP)	2021 [153]
Reversing sintering effect of Ni particles on γ-Mo_2_N via strong metal support interaction	Atomic redispersion of Ni NPs on γ-Mo_2_N induced by H_2_/N_2_	H_2_, N_2_; 400–630 °C, 2 Pa	MEMS-based ECELL	2021 [46]

DP, with differential pumping system; CAEM, controlled atmosphere electron microscopy; ECELL, environmental cell

^a^ VHPO, VOHPO_4_ 1/2H_2_O; VPO, (VO)_2_P.

The present reports can be grouped in 4 categories: (a) visualization of single atoms under controlled temperature and gas environment conditions, (b) visualization of the response of suspected active sites/phases to chemical environments, (c) visualization of a chemical process, and (d) investigation of a catalytic reaction.

### Visualization of single atoms under controlled temperature and gas environment conditions

**Fig. 7.**
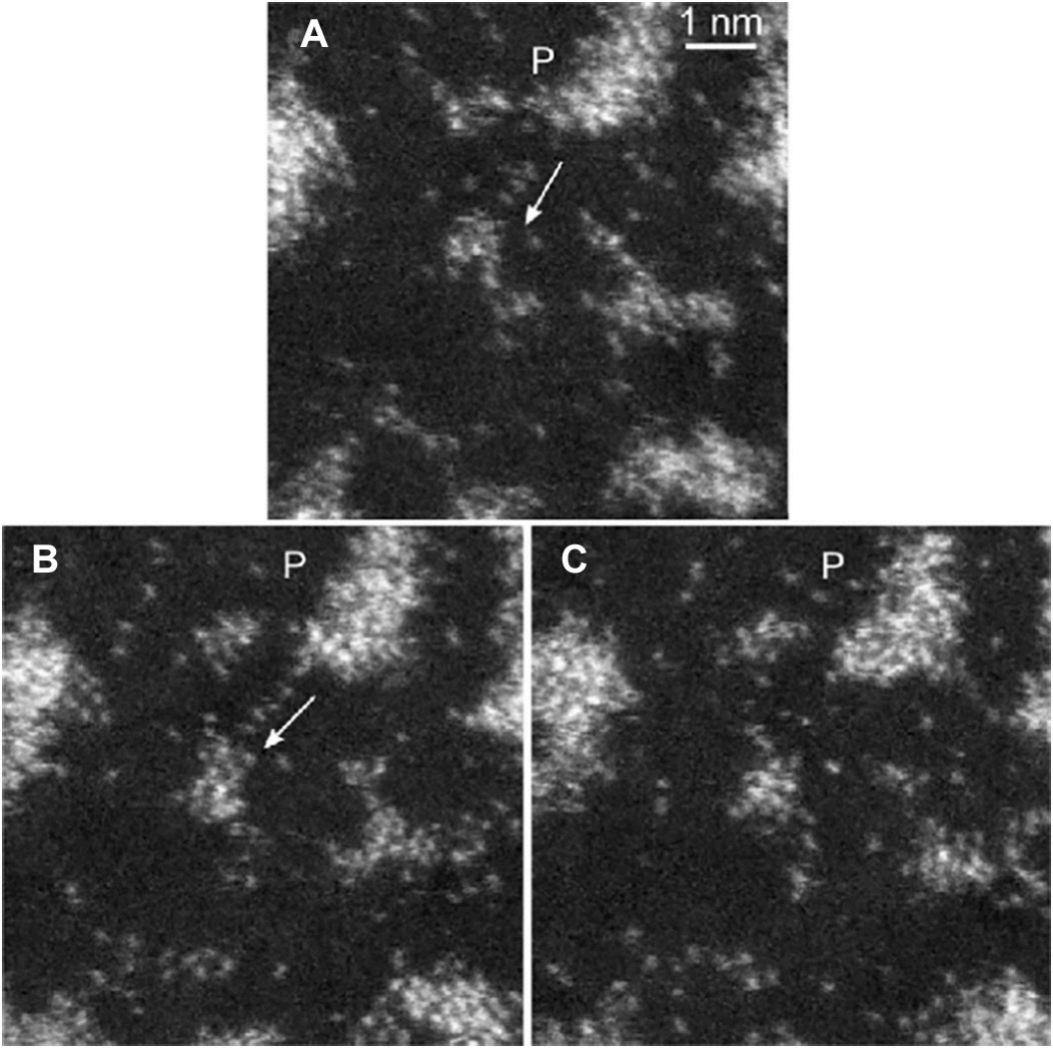
Dynamic process of single atoms in Pt/C catalysts: migration of single atoms [e.g., the atoms arrowed in (A) and (B) from the NP P], resulting in increased faceting of the NP and the formation of clusters (C). Reprinted with permission from [74].

Single atoms and clusters consisting of a few atoms play a critical role in supported catalysts, where they are suspected to act as active sites in catalytic reactions [73]. Single atoms on support have been imaged already in 1980s by Crewe on a dedicated STEM. In the past years, with probe-corrected STEM, the images of single atoms can be routinely obtained and have been used as evidence for so-called “single atom catalysis.” However, the images are usually obtained in high vacuum. It is of course important to know whether such single atoms are also existing in a given chemical environment. Boyes and Gai developed aberration-corrected ESTEM (AC-ESTEM) and analyzed particle forms and single atom number density under 0.01 to >0.1 mbar gas pressure (e.g., H_2_ and O_2_) at temperature from 25 to ~500 °C [57,59,74]. They also studied the mobility of single atoms on the support (Fig. 7) and found that the NPs act as both sources of potential ad-atoms/clusters and recipients for them, and low-coordination atoms on the surface are substituted with surface facets by structural rearrangement minimizing surface energy. All of these dynamic structural features are evidences to relate the often-discussed Ostwald ripening to deactivation of catalysts. It remains to be seen if these observations really relate to active sites: The fact that these species are sufficiently beam stable for observation and the inability to see light atoms forming compounds with the heavy atoms detected render it unclear if such features are truly “high energy” sites or possibly mere spectators [75] that are involved in reshaping particles but not directly in catalytic transformations.

### Visualization of the response of suspect active sites/phases to chemical environments

**Fig. 8.**
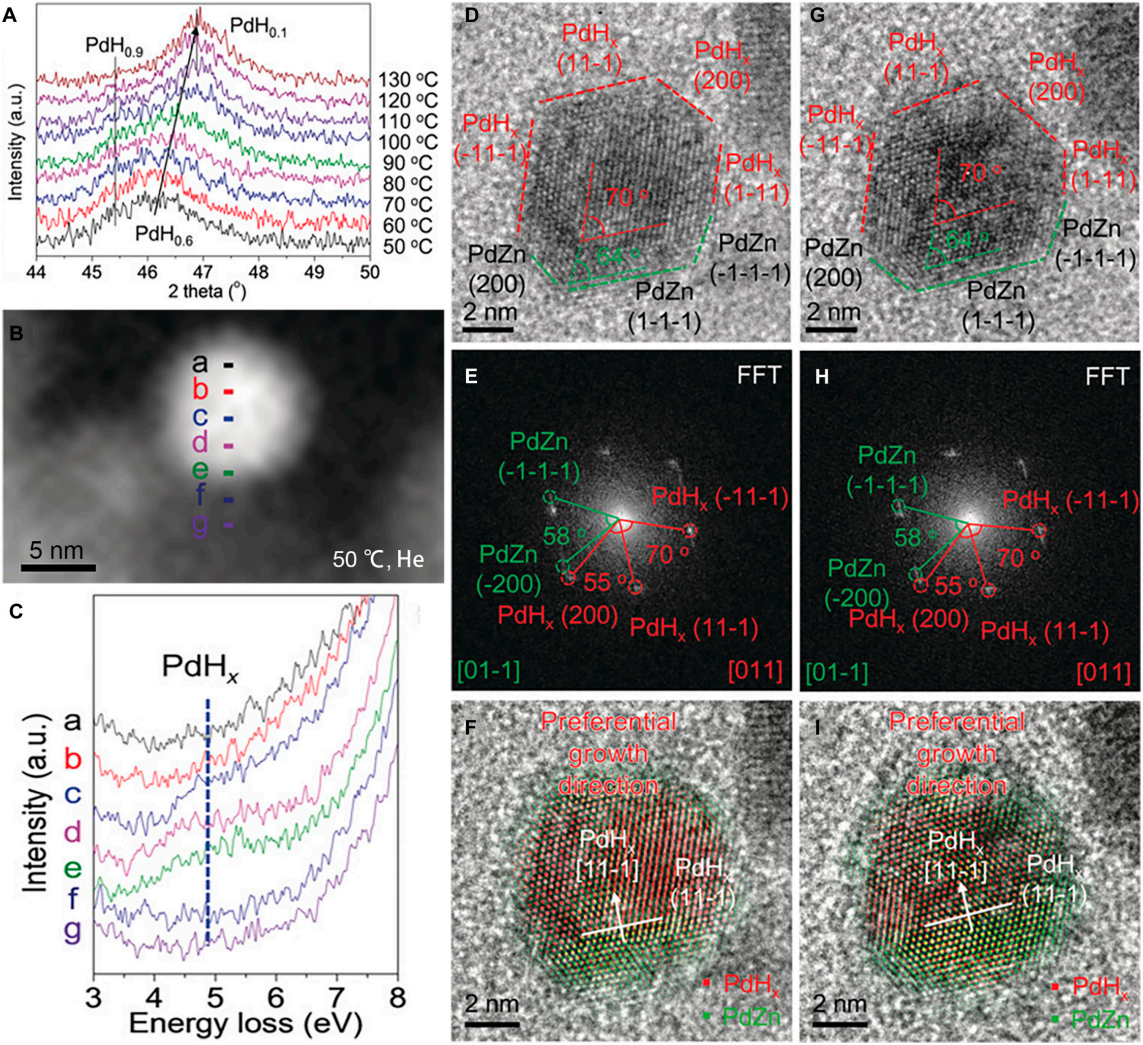
(A) In situ XRD profiles of PdH*_x_* species at different temperatures ranging from 50 to 130 °C. (B) HAADF-STEM image of a Pd/ZnO catalyst and (C) its corresponding EELS spectrum. HRTEM images of a Pd/ZnO catalyst maintained at 300 °C under an H_2_ atmosphere for (D) 20 min and (G) 40 min. (E) and (H) are local fast Fourier transforms (FFTs) that correspond to (D) and (G), respectively. (F) and (I) are inverse FFTs from (E) and (H), respectively. Reprinted with permission from [76].

CEM provides the unique opportunity to explore and illustrate the dynamic active phase under controlled chemical environments, which cannot be identified ex situ due to its instability. Niu et al. [76] studied the formation process of ZnO-supported PdZn catalyst under H_2_ atmosphere using window-type ECELL tools. As indicated by the in situ XRD data, an intermediate phase PdHx was first generated at a relatively low temperature before the formation of PdZn (Fig. 8A). Both the in situ TEM imaging and in situ STEM-EELS analysis verify the formation of hydride phase at 50 °C under H_2_ atmosphere (Fig. 8B and C). Afterward, the following phase transition from PdH*_x_* to PdZn was visualized at atomic resolution under the realistic chemical conditions, rationalizing that interstitialization occurs in the interface area and subsequently PdZn grown along PdH*_x_* <111>, thus causing the PdZn projection area to expand (Fig. 8D to I).

**Fig. 9.**
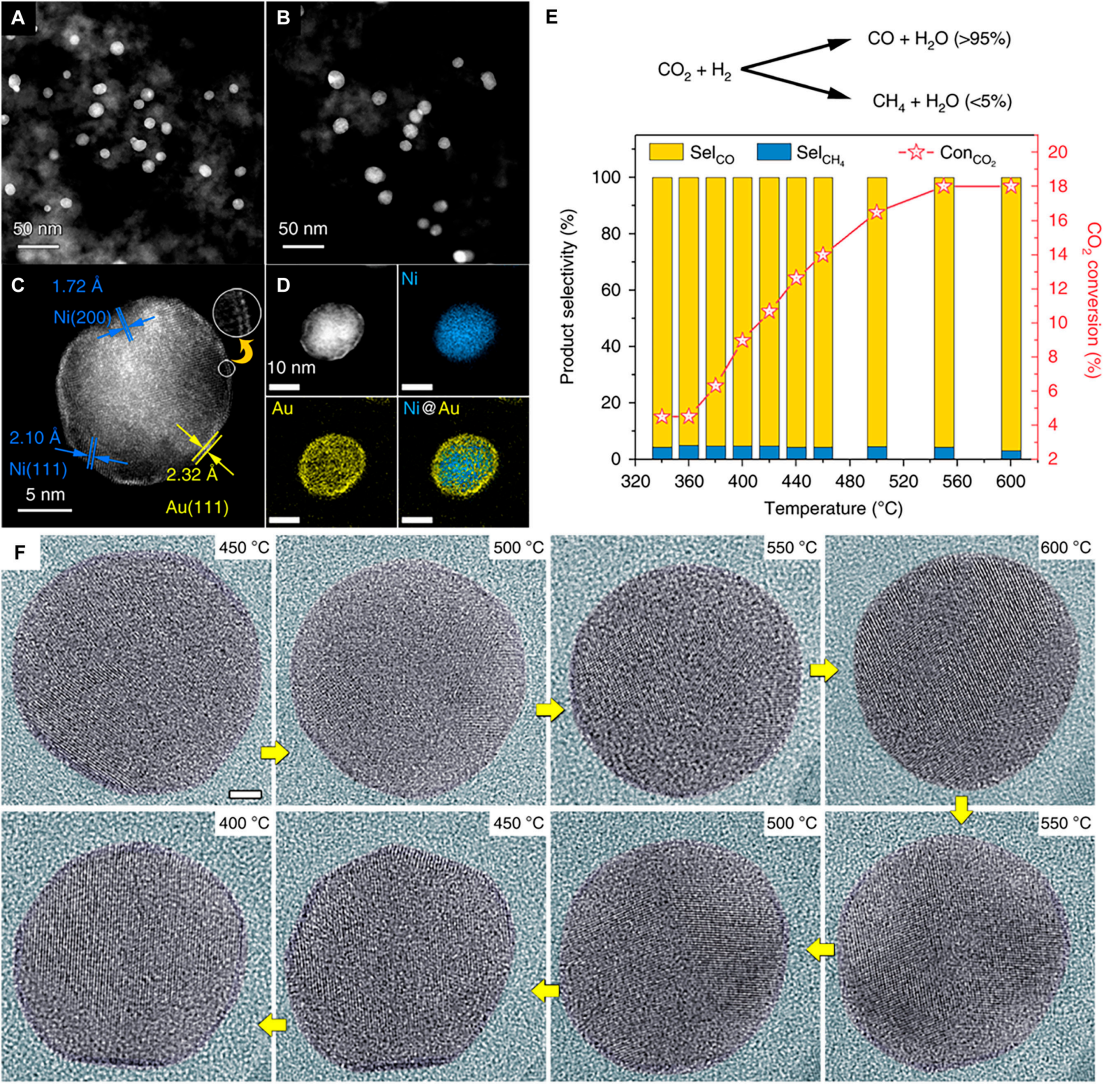
HAADF-STEM images of Ni@Au nanoparticles (A) before and (B) after CO_2_ hydrogenation reaction. (C) and (D) are atom-resolved HAADF image of a Ni@Au NP after CO_2_ hydrogenation reaction and its elemental distribution analysis, respectively. (E) Catalytic performance of Ni@Au/SiO_2_ for CO_2_ hydrogenation (~1 atm, 24 volume % CO_2_ + 72 volume % H_2_ + 4 volume % Ar at a space velocity of ~60,000 ml g^−1^ h^−1^). (F) In situ TEM imaging of alloying and dealloying evolution of a single Ni@Au NP during CO_2_ hydrogenation reaction. Reprinted with permission from [77].

Liu et al. further exemplify the microstructural characteristic of the type IV catalyst by using in situ TEM tools to study the structural evolution of Ni@Au core–shell NPs during CO_2_ hydrogenation reaction. Their work discloses that the reconstructed surface alloy forms during the reaction conditions but resegregates into the core–shell structure after the removal of reactants (Fig. 9) [77]. Clearly, based on ex situ data of the fresh and used catalysts, one may consume that the high catalytic performance should ascribe to the outer Au shell since the Au shell remains unchanged before and after the reaction. However, the observed alloying/dealloying process with respect to the different environments indicates that the dynamic formed mixed NiAu alloy should play a key role in the hydrogenation reaction. This work challenges the general assumption that the core–shell structure remains in its configuration under experimental conditions, and inspires us to reexamine the active phase and structure–activity relationship of similar binary catalysts.

Takeda et al. studied CO oxidation over Au/CeO_2_ at room temperature in CEM [78]. CO molecules are adsorbed on Au NP surface and maintain the shape of Au NPs enclosed with {111} and {100} planes. Partly aiding from electron irradiation, oxygen molecules can be dissociated at the perimeter interface between Au NP and CeO_2_ support. Zugic et al. [79] found the surface restructure of nanoporous Au–Ag alloys after ozone pretreatment, which created a silver-rich oxide layer. The amorphous oxide layer could be removed by CO, and consequently, oxygen-stabilized Ag–Au alloy sites were found. It indicates that catalysts can exhibit highly dynamic geometrical and compositional changes during both activation and reaction conditions. In a different study, Hansen et al. identified that the shape of zinc oxide supported copper nanocrystal changes with different gas environments (H_2_, H_2_ and H_2_O, CO and H_2_O) at a pressure of 1.5 or 5 mbar at 220 °C. This account of structural dynamics responding to changes in chemical potential reveals the strong influence of chemisorption on the shape and structure of NPs far below the melting point of the bulk phase [80]. Yuan et al. [81] also reported the dissociation of H_2_O over anatase TiO_2_ (001) surface and the consequent formation of twin-protrusion structure due to the adsorption of hydroxyl species. Dong et al. [82] visualized the periodic surface reconfigurations of the CuAu surface upon CO and H_2_O gas mixture exposure by using ETEM, which contrasts with the surface segregation and oxidation induced by CO and H_2_O gas, respectively. These reports warns us about premature conclusions on the morphology and surface structure of nanostructured catalysts in operating conditions. The details of the adsorbate–particle interaction on one hand and of the particle–support interaction (including local defects) on the other hand control the structural evolution. The multiple predictions from theory on the equilibrium morphology of NPs are valid under their conditions but may be misleading when extrapolated to reactive conditions.

**Fig. 10.**
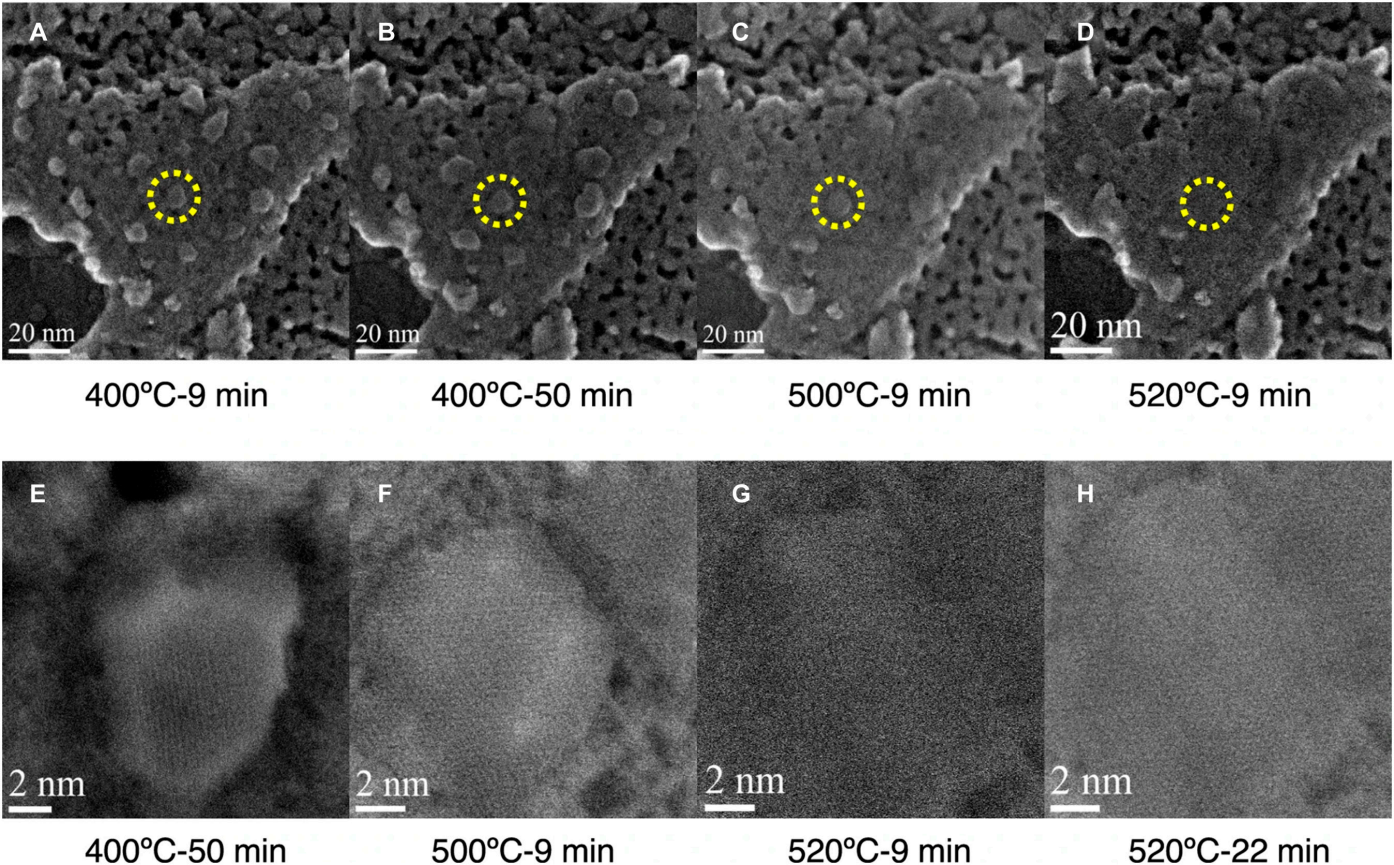
(A to D) Low-magnification STEM-SE images of γ-Mo_2_N-supported Ni particles in H_2_/N_2_ at 400 °C for 9 min, 400 °C for 50 min, 500 °C for 9 min, and 520 °C for 9 min, respectively. (E to G) High-magnification STEM-SE images of selected region (marked as yellow circle in (B) to (D). (H) High-magnification STEM-SE image of Ni-4nm/γ-Mo_2_N catalyst at 520 °C for 22 min. Reprinted with permission from [46].

In principle, the surface atom configurations of supported NP and/or interface between NP and support play a key role in heterogeneous catalysis. However, it is not possible to simply extract 3D topographic information from a 2D TEM or STEM image. To directly visualize surface atom configuration and morphology, advanced techniques, like atomically resolved secondary electron (STEM-SE) imaging [45], atomic-resolution electron tomography (single particle reconstruction), and quantitative electron tomography [83], have been deployed under static conditions and high vacuum. It is of great interest to develop these methods to measure evolution of surface structure and/or morphology at atomic scales and under realistic reaction conditions to provide details related with catalyst activation, deactivation, and other side reactions. Bals et al. obtained a series of 3D images of a single Pt NPs via reconstruction of corresponding HAADF-STEM images captured during various oxidation−reduction cycles. This work demonstrates that the oxidative treatment could induce the formation of high-index facets, which disappeared after exposure to the reductive environment [84]. Liu et al. first visualized the reconstruction and redispersion of Ni NPs into atomic deposition on Mo_2_N using in situ atomic resolved secondary electron imaging technique (Fig. 10). The structural transition disclosed in this work could be due to the strong metal–support interaction modulated by the gaseous stimulus under the chemical environment [46,85,86].

### Visualization of a chemical process

One advantage of CEM is that it allows the recording of chemical process when a solid reactant is involved [87]. Examples are the growth of metal NPs or of carbon nanotubes at liquid–solid or gas–solid interfaces, respectively. Liao et al. [88] observed the Pt_3_Fe nanorod growth from NP building blocks and established the mechanism of 1D colloidal nanocrystal growth. Zheng et al. visualized the dynamic processes of the metal (In, Sn) solid–liquid interface at atomic scale in real time and identified a quasi-liquid phase of 2 to 3 nm at the interface of a metal NP and aqueous solution. The metal clusters can be nucleated and grown into NPs directly from the quasi-liquid phase [89]. Helveg et al. [90] presented the growth process of carbon nanotubes from CH_4_ decomposition over supported nickel catalysts. Combined with density functional theory calculations, the results indicate that the metallic step edges act as growth sites for graphene growth.

The chemical process of oxidation and reduction on the topmost surface of the catalysts can also be captured in CEM [60,91,92]. One example is the oxidation reaction of cobalt-based catalysts studied in an ECELL (pressure, 1 to 4 bar; temperature, 95 to 400 °C) [93]. Based on the kinetic data, the diffusion coefficient of Co in the cobalt oxide shell was estimated to reveal the diffusion pathway and to uncover the evolution of the Kirkendall effect. Furthermore, Han et al. [94] used STEM-EELS tomography technique to quantitatively image the oxidation-induced chemical segregation of CoNi nanoalloy. Another example is the redox process at the surface of Pt NPs supported on CeO_2_ in reactive gases (O_2_, CO, and H_2_O vapor). AC-ETEM shows the evolution of the atomic structure of Pt oxide on the Pt NP surface in O_2_ at room temperature. The surface Pt oxides are reduced to Pt in both vacuum and CO atmosphere [95]. It was also identified that H_2_O vapor can suppress the oxidation of the Pt surface. It is informative to compare these findings on nanostructures to those of combined scanning tunneling microscopy (STM)/sum frequency generation (SFG) studies of the interaction of CO with extended surfaces [96–98] of Pt. Here, it was found that clean CO did not do any structural change to Pt, whereas contaminated (with possibly water) Co did strongly restructure CO and led to Pt carbonyl surface compounds. The results indicate that the observation of atomically resolved structural changes at elevated pressures might critically depend upon the exact nature of the chemical potential. This is barely controlled in any CEM experiment today.

Sintering is a crucial factor for deactivation for many supported metal catalysts in the reactions carried out at high temperature or under severe conditions [99]. The involved mechanism at atomic level (i.e., Ostwald ripening or particle migration and coalescence) can be systematically studied using CEM [100]. Simonsen et al. [101] studied the dynamic sintering process of Pt NPs supported on a planar SiO_2_ in 10 mbar synthetic air at 650 °C, showing the time-resolved evolution of particle size distribution (PSD) and of the individual Pt NP growth or decay. In situ investigations reveal that the sintering is dominated by Oswald ripening. Growth rates, PSD, and particle sizes are in line with kinetic models that originated from mean field assumption. For the individual Pt NPs, the ripening process is governed by the size and location of the adjacent NPs. In addition, the dynamic growth and agglomeration of NPs in solution were also investigated using liquid cell TEM [102–104]. Tan et al. [105] reported the real-time TEM imaging of the overgrowth process of Ag on Au nanocubes in solution. The observations revealed the Ag NPs attached onto Au nanocubes formed homogeneous and conformal Ag shells via coalescence, resulting in an Au–Ag core–shell nanostructures. The Ag atoms absorbed directly onto Au nanocubes via monomer attachment could also lead to the same Au–Ag core–shell NPs. Dachraoui et al. [106] used in situ liquid cell TEM to observe the 3 different coalescence processes that carry the growth and nanocrystallization of Pd-Pt bimetallic alloys in aqueous solution: amorphous–amorphous, amorphous–crystalline, and crystalline–crystalline coalescence. These findings are essential for the understanding of the mechanism of the growth and agglomeration of NPs in liquid environments and shed light on the further rational design of liquid-phase catalysts.

Electrochemical lithiation occurring in the solid-state electrodes is a key process for exploring the lifetime of anodes in lithium-ion batteries [107–109]. In situ observation of electrochemical lithiation in silicon at atomic scale has been studied in a special TEM holder designed by Huang et al. It occurs that the lithiation kinetics are controlled by a ledge mechanism, i.e., the lateral movement of ledges on the close-packed {111} planes. The amorphous Li*_x_*Si is produced from layer by layer peeling of the {111} facets, uncovering the mechanism of amorphization and the orientation-dependent mobilities of the interface. These studies are the observations with electrolytes, the solid-electrolyte interphase, and the reaction products of decomposing salt, which are far from the real cell operation. In addition, the possible beam artifacts and the defect structure of the Si used need attention. Zeng et al. developed an electrochemical liquid cell for in situ liquid cell TEM by using photolithography that enables visualizing and recording the complex electrochemical reactions at nanoscale in real time, including lithium dendritic growth, solid electrolyte interface formation, and electrochemical lithiation [110]. It provides a powerful research tool for the fundamental understanding of the electrochemical reactions associated with energy devices.

### Investigation of a catalytic reaction

Due to the small amounts of catalyst in an ECELL, the analysis of reaction products is usually measured with more material in an ex situ microreactor under similar conditions for exploring the structure–function relationship. Usually, the samples were placed on a TEM grid for the following TEM study on the same catalysts. This is the recently developed identical location TEM [111], which allows one to track changes of catalysts before and after catalytic reaction and mostly used for electrochemical reactions [112–117]. However, it is hardly used to study time-resolved evolution of active sites at the solid–liquid–gas interfaces since there might be some changes when a sample was removed from native environment and placed under a high vacuum in electron microscopes. To overcome this deficiency and study the catalytic reaction in CEM, operando technique has been developed. This term, operando, very often used in the field of Raman spectroscopy, which was proposed by Bañares, Weckhuysen, and others as an alternative for in situ spectroscopy to emphasize the structure and catalytic products, is detected simultaneously without using the ex situ studies [118–122]. The operando approach identifies the changes in the structure and composition of the catalysts undergoing catalytic reactions and reveals how the gas molecules interact with the catalyst surface/interface, which is important for establishing structure–performance relationships in real time [123]. For instance, Vendelbo et al. [124] presented a representative example that synchronously shows the atomic dynamic refaceting of Pt NP and corresponding performance during oscillatory CO oxidation by using a nanoreactor in ETEM, mass spectrometry (MS), and calorimetry. The oscillations have been explained reasonably based on the analysis of dynamic properties. Here, the nanoreactor includes a unidirectional gas flow channel with a reaction zone at 1 bar pressure and elevated temperature. In addition, the strategy of in situ gas-phase TEM coupled with online MS could be used to analyze a trace of various gas products [125], which is tremendously valuable for many catalytic reactions, such as CO oxidation [126] and CO_2_ hydrogenation [127]. The combination enables the investigation of the structural evolution of catalysts while simultaneously resolving the reaction properties, which helps to understand the related reaction mechanism and rationalizes the structure–activity correlations at an atomic scale.

Apart from MS, EELS is becoming a tool for directly measuring the catalytic products in ECELL, as the products can be detected in the reaction volume without the complication of transferring and diluting them in a mass spectrometer. The method works well for small molecules as reactants and products, in cases of more complex molecules the low-loss region of EELS spectrum where molecular vibrations can be resolved and further discriminated the educts and products. The core loss region is suitable when hetero-nuclear reactions such as oxidation or halogenation of hydrocarbons are the reaction of interest. An example from the literature concerns the detection of the reaction products from CO oxidation and from CO_2_ methanation over Ru/SiO_2_ catalysts [120]. Lu et al. [128] also used EELS to detect the production of H_2_ during the photocatalytic water splitting and the accompanying reduction of TiO_2_ surface shell. Recently, it has been reported that the energy resolution in EELS can reach 10 meV [129], which will help to overcome the peak overlap and improve the capability to analyze complex reactions. There remains a challenge with the sensitivity of EELS. A simple solution to this limitation is to increase the amount of catalyst present in the area of sight of the TEM grid that are exposed to the identical reaction conditions in the TEM sample holder. A method is described for preparing TEM samples consisting of a catalyst pellet carrying a minute specimen of the identical material in a central bore of the pellet [130]. Both MS and EELS were used to calculate catalytic conversion, while simultaneous images of the atomic structure were acquired [131].

## What We Can Know Better with CEM

The CEM methodology is still in its infancy. The practical difficulty of using it and the still substantial efforts for creating a useful reaction environment at the high-resolution analytical TEM/EELS instrument that is required for meaningful observation will disappear if industry provides a better integrated instrumentation. This will only occur if sufficient application potential pays back for the development of dedicated CEM instrumentation. Having such instrumentation is, on the other hand, a prerequisite for broader dissemination of the method among chemists who do not wish to modify or put at risk advanced (multi-purpose) electron microscopes.

All this will not happen if CEM will only deliver beautiful images of reacting solids for special show-case examples, confirming the understanding that we can reach also with other analytical methods. An “add-on” method to conventional TEM in catalysis [1] would not be sufficient to drive the development of CEM into a central tool of modern catalysis science and a “must have” method for all those being concerned with the development of high-performance catalysts based on rational approaches.

The authors wish to convey the notion that exactly that is the case with CEM. If we wish to bring the rational approach or the “design approach” in heterogeneous catalysis to finally a real application, then we need structure–function correlations that describe causal relations between real surface structures of working catalysts. Only then can we expect that these relations are predictive and can be extrapolated to either other reaction conditions or other chemical structures. The advent of broadly based scaling relations [132–134] between catalytic reactivity and surface electronic properties that are based on a theoretical concept give us for the first time the hope that such an approach may be realized. The verification of the validity of the approach [135,136] is still circumstantial as the systems made after the predictions form theory are not analyzed for the causal origin of their catalytic performance. Comparing relevant observable structural features (in situ) other than difficult to assess rates with the respective properties predicted from theory may be a strategy for achieving this verification.

Here comes CEM into play. This suite of methods for deriving simultaneous catalytic function and several geometric and electronic structural features that are amenable for theoretical prediction is a unique way of approaching the causal relation of theory and experiment in a highly integrated practical approach. In addition, CEM is likewise highly descriptive methodology supporting the synthesis of novel catalyst systems as minute sample amounts of the required nanostructures carrying the desired functions [20,137,138] can give a whole array of functional and structural information. This exciting prospect does not devaluate the more conventional suite of in situ analytical techniques, but it can act as a versatile and rapid complement in early stages of catalyst development projects.

CEM is additionally an invaluable tool in defining our understanding of prerequisites for suitable catalyst materials. As we do not need model systems for observing working surfaces and we can relax assumptions about static surface structures as we can observe atomic details in real time, we gain a realistic view upon the dynamics [139–144] of catalytic materials under truly “near-ambient” conditions. It is mostly the unknown residence time and transport property distribution that make CEM observations still an approximation to a real catalytic operation in an optimized reactor environment. As, however, a CEM experiment delivers a multitude of observables (conversion and selectivity, EELS-NEXAFS (near-edge x-ray absorption fine structure) data, geometric and phase structural information, surface compositions, data on deactivation), it is not too difficult to scale these information with other in situ methods observing larger amounts of catalyst and finally arrive at a kinetic description allowing to bridge to the real reactor observations.

## The Challenges and Future Development of CEM

**Fig. 11.**
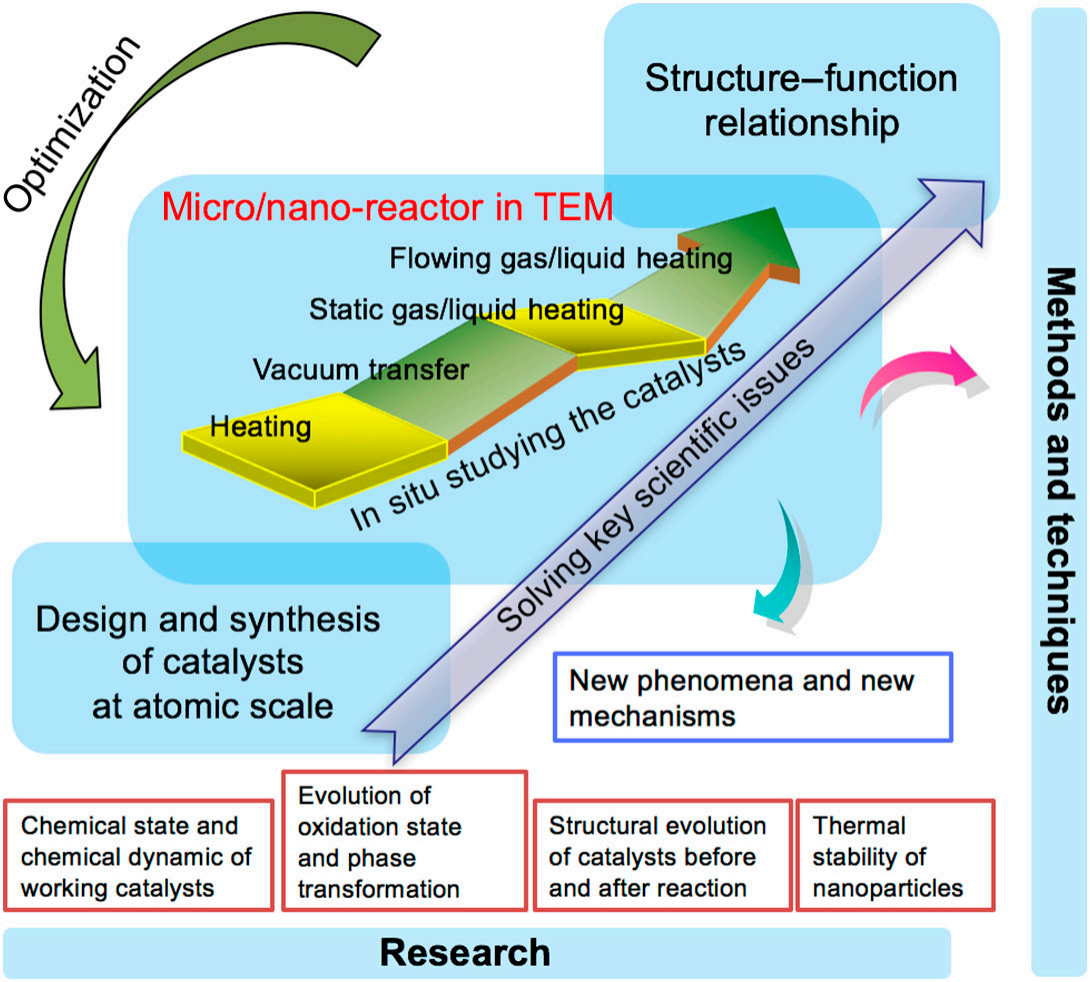
The development roadmap of CEM on the research contents of catalysis and the innovation of methods and techniques (e.g., special TEM holders).

CEM as a multifunctional toolbox has made some contributions for understanding some process of nanoscience, especially in catalysis science. As shown in Fig. 11, the methods and techniques have been innovated from heating holder to static/flowing gas/liquid-heating holder or differential pumped microscope that mimics reaction environment. The research possibilities have been extended from thermal stability of NPs to dynamic structural evolution of working catalysts. It remains challenging to measure the activity and selectivity of the catalyst simultaneously with the atomic-scale surface/interface structure so that the complete structure–function relation could be directly established in various catalytic reactions. Revolutionary advances in CEM in the future are expected to be implemented step by step.

### Environment around catalysts in CEM

There is a small quantity of residual gases in CEM, although the vacuum in it is about 10^−8^ to 10^−6^ mbar. These gases may react with, absorb on, or contaminate the top surface of catalysts, so the gas composition must be identified by MS and the affection of residual gases on the catalysts should be tested. In addition, the reactant gas should not react with any parts of CEM. The pristine environment in window-type ECELL is better than that in differential pumping-type ECELL, but the membranes of windowed ECELL should be inactive to catalytic reaction. Important developments are required both to study the reactivity of today’s combinations of materials in ETEM and in ECELL systems and from there to identify minimum reactivity materials that still fulfill the stringent requirements of high-resolution electron microscopy.

The detection methods for reaction products need further massive improvements. This concerns the materials and dimensions of the tubing, bringing products to analysis and the analytical instrumentation by itself. The performance of modern GC instruments is well adequate for many systems if more work is dedicated to the method development. In addition, the family of ion–molecule reaction mass spectrometers allows for unsurpassed detection limits in the part per trillion (ppt) range with real-time resolution and can be quantified for deriving useful catalytic data.

Electron irradiation is an inevitable but crucial issue in CEM. Controlled electron irradiation can be used to stimulate some chemical processes at the nanoscale, providing opportunities for fabrication of interesting nanostructures. One example is the use of electron beam-induced localized surface plasmons in Al NPs to initiate graphite etching, which facilitates the reduction of CO_2_ to CO by carbon at room temperature [145]. However, the effect from electron irradiation in a chemical environment at elevated temperature could be worse and must be ruled out for any reliable experiment. For instance, the electron beam can ionize oxygen molecules into atomic and ionized oxygen [146]. Creating defects, changing the PSD of supported catalysts, and destroying the long-range ordering of a crystallize catalyst could be faster and worse in a CEM. The observation with low electron beam dose without invasive exposes and the comparative and repetitive experiments are needed. The abovementioned contents will minimize the influence of environment and avoid misunderstanding or wrong interpretation of experimental data.

### Extending the category of catalytic reaction

Usually, CO oxidation was selected as the probe reaction in a CEM because its mechanism is well understood and the gases are secure to CEM. An expansion of gas types, including corrosive and condensable gases, is expected for studying more reactions. The development of liquid ECELL is essential to understand the growth mechanism of catalysts and the dynamics in liquid-phase and electrochemical reactions. The interaction between electron beam and sample should be evaluated and quantitative analysis, which is helpful for probing the natural of heterogeneous catalysis and revealing the corresponding intrinsic mechanism.

To achieve the above, it seems essential to pay more attention to the transport properties of CEM reactors. Most “complicated” reactions exhibit severe limitations in selectivity and conversion under ill-defined mass transport conditions. This can easily lead to erroneous results when comparing CEM results with expectations form large-scale kinetic testing (in laboratory “microreactors”) or from theory. The remedy against this is the adaptation of the testing conditions in CEM to the respective transport conditions in the used nanoreactor. This can be done by operating a versatile gas handling system (more advanced than presently available with TEM instrumentations) and by knowing the transport characteristic of both the sample and the reactor. To gain these information on routine basis, it seems essential to develop a reaction system standing in parallel to the microscope that allows for predetermining suitable reaction parameters before inserting the sample into the electron microscope. By comparing the performance with and without beam obtained from the 2 experiments, we can finally also assess convincingly the absence of beam-induced artifacts on the data that ultimately may limit the application of CEM as a versatile and universal catalyst testing instrument.

Another crucial challenge is correlating the changes in the catalysts in working conditions with the reaction products in real time. MS can give some valuable information in CO oxidation [124] and redox of metal catalysts [147], but more products that passed through the nanoreactor need to be collected and identified in more catalytic reactions. It needs high-resolution MS and chromatographic analysis, overcoming the small change of atmosphere caused by less catalysts participating in the reaction.

### Developing and updating the hardware and software in CEM

Improving and updating of spectroscopy techniques in CEM enables to explore more chemical information and understand the electronic structure of catalysts, such as improving the sensitivity of EDX and equipping the detectors of Raman, XPS, and IR. With these equipments, CEM will become a chemical analysis toolbox that can accurately identify the electron interaction, valence state, heteroatom doping position, species of functional groups, etc. However, these characterization techniques will suffer from a problem, that is, tiny amount of materials and not enough signal. The special nanoreactor with sufficient catalysts or parallel and simultaneous micro-reflections should be designed to realize the imaging and spectroscopy characterizations.

The other hardware and software of user interface are also expected to be improved. For instance, the highly sensitive and ultrafast time-resolved resolution charge-coupled device recording system needs to be installed on CEM for quantitative measurements of surface/interface structure, composition, and bonding evolution of catalyst in relevant environments; thus, the structure–function relationship at the atomic or molecular level with high spatial resolution and high temporal resolution can be achieved. In general, the commercial CEM (including imaging, spectroscopy, and electron diffraction), which usually enables readout speeds of thousands of frames per second, provides the opportunities to explore many dynamic features under the conditions far from equilibrium. In addition, adding some automated adjustment (e.g., focus and astigmatism) and self-running operations in user interface enables CEM optimal handing and easy generating data.

## Summary

By combining imaging and spectroscopy techniques, the CEM becomes an innovative and powerful toolbox and is used to capture and analyze the chemical state and chemical dynamic of working catalysts. The differential pumped ETEM, windowed ECELL, and operando are useful and effective approaches for unraveling gas–surface or liquid–surface phenomena under controlled environment in heterogeneous catalysis and NP research. They help to reveal the geometric and electronic structures, understand the dynamics and kinetics of catalyst transformation, and address structure–function relations. With the development of CEM and associate techniques, the mechanisms of catalytic reactions will be established. These will support the historic quest for a design of novel catalytic materials. For those suitable targeted synthesis methods, allowing to predetermine the outcome of the reaction will be required. Our current methodological portfolio is less suited for such target synthesis. To improve on that situation, CEM may also be helpful as multiple synthesis unit operation (e.g., thermal processing) can be studied under real conditions. CEM will thus help to evolve nanoscience and nanotechnology. However, the development of CEM will meet a lot of unforeseen difficulties; it needs the cooperation of scientists on material, microscopy, spectroscopy, and chemistry together with experts in hardware development both from academic institutions supporting long-term developments and from industry, making the results of prototype developments available to the wider community.
